# Genomic Medicine in Periodontal Disease: Old Issue, New Insights

**DOI:** 10.1177/08987564221109102

**Published:** 2022-06-28

**Authors:** Nuno Gonçalves-Anjo, João Requicha, Andreia Teixeira, Isabel Dias, Carlos Viegas, Estela Bastos

**Affiliations:** 1 Department of Genetics and Biotechnology, School of Life and Environmental Sciences, 56066 University of Trás-os-Montes e Alto Douro (UTAD), Vila Real, Portugal; 2 Centre of the Research and Technology of Agro-Environmental and Biological Sciences (CITAB), Institute for Innovation, Capacity Building and Sustainability of Agri-food Production (Inov4Agro), UTAD, Vila Real, Portugal; 3 511313 Department of Veterinary Sciences, School of Agrarian and Veterinary Sciences, UTAD, Vila Real, Portugal.; 4 Animal Research Centre (CECAV), UTAD, Vila Real, Portugal; 5 Associate Laboratory for Animal and Veterinary Sciences (AL4AnimalS), Portugal

**Keywords:** periodontal disease, *Canis lupus familiaris*, genomic medicine, *RANK*, TNFRSF11A, polymorphisms

## Abstract

Genetic variability is the main cause of phenotypic variation. Some variants may be associated with several diseases and can be used as risk biomarkers, identifying animals with higher susceptibility to develop the pathology. Genomic medicine uses this genetic information for risk calculation, clinical diagnosis and prognosis, allowing the implementation of more effective preventive strategies and/or personalized therapies. Periodontal disease (PD) is the inflammation of the periodontium induced mainly by bacterial plaque and is the leading cause of tooth loss. Microbial factors are responsible for the PD initiation; however, several studies support the genetic influence on the PD progression. The main purpose of the present publication is to highlight the main steps involved in the genomic medicine applied to veterinary patients, describing the flowchart from the characterization of the genetic variants to the identification of potential associations with specific clinical data. After investigating which genes might potentially be implicated in canine PD, the *RANK* gene, involved in the regulation of osteoclastogenesis, was selected to illustrate this approach. A case-control study was performed using DNA samples from a population of 90 dogs – 50 being healthy and 40 with PD. This analysis allowed for the discovery of four new intronic variations that were banked in GenBank (g.85A>G, g.151G>T, g.268A>G and g.492T>C). The results of this study are not intended to be applied exclusively to PD. On the contrary, this genetic information is intended to be used by other researchers as a foundation for the development of multiple applications in the veterinary clinical field.

## Introduction

Periodontal disease (PD) is the most prevalent condition in the oral cavity of companion animals.^1,2^ PD is a progressive infection-driven inflammatory disease of tooth-supporting tissues termed, 'the periodontium'.^3^ PD manifests initially as gingivitis, which is its reversible form, and can progress to periodontitis, the chronic and irreversible stage, characterized by alveolar bone resorption and destruction of the periodontal ligament and root cementum.^4,5^ When not properly treated, PD can lead to loss of periodontal tissues and tooth exfoliation.^6^ In addition, periodontitis is associated with many chronic diseases and conditions affecting general health.^3^

The first cases of canine PD, to the authors’ knowledge, was reported in 1899, when 75% of stray dogs were described to have pyorrhea alveolaris.^7^ Further studies observed severe calculus deposits and gingival inflammation in 95% of Beagle breed dogs older than 26 months of age.^8^ In Poodles, 90% under 4 years of age and all dogs older than 4 years old were documented to have, at least, one tooth with periodontitis.^9^ In 2005, one study stated that PD was present in 40% of dogs aged from 1 to 4 years and in 89.4% of dogs aged from 12 to 13 years.^10^ Other authors stated that PD has a higher prevalence in small and miniature breeds.^1^ More recently, a study in Miniature Schnauzers showed that the severity of gingivitis in periodontitis affected teeth was variable and that the periodontitis progression rate was significantly faster in older dogs.^11^ In a population of Labrador retrievers aged 1.1 to 5.9 years, all dogs were found to have gingivitis at the initial assessment and, after 2 years, 56.6% of dogs developed periodontitis with a significant positive correlation between the proportion of teeth with periodontitis and age.^12^

Canine PD is a multifactorial disease. The main etiological factor is bacterial biofilm formation, however behaviural, environmental, genetic and epigenetic factors can facilitate disease development. Thus, the clinical phenotype results from an interplay of these factors.^4^ The focus of the research in the last decade has been to find the major determinant factors of the inflammatory mechanism. In contrast to humans, it has been demonstrated that plaque in the healthy dog was dominated by Gram negative bacterial species, whereas Gram positive anaerobic species predominated in dogs affected by PD.^13^ PD is mainly caused by the accumulation of bacterial plaque on teeth and the gingival margin and through their direct and indirect toxins that trigger an immune response. However, genetic predisposition seems to have an important role. In humans, the heritability is estimated to be greater than 50%.^4^ Thus, it is important that clinicians understand some basic concepts of genetics.

The genome is the genetic material of an organism, including coding and non-coding regions from the nuclear DNA (nDNA) and the mitochondrial DNA (mtDNA). mtDNA encodes ribosomal RNA (rRNA), transfer RNA (tRNA) and proteins involved in the oxidative phosphorylation process.^14,15^ A gene is a sequence of DNA which contains information to encode synthesis of a functional product, such as a protein or a regulatory RNA.^16^ The structure of a gene comprises many elements, such as: the enhancer/silencer, the promoter and the 5′ and 3′ untranslated regions (UTRs) known as regulatory sequence, and the open reading frame – composed of exons and introns – which is often a small part of a gene. The regulatory sequence plays an important role, controlling when and where expression occurs. During transcription, the gene is transcribed into a pre-messenger RNA (pre-mRNA) which contains UTRs at both ends as well as exons and introns. Then, the maturation of the pre-mRNA occurs, and the introns are removed (splicing process), resulting in a mature messenger RNA (mRNA) composed of a protein coding sequence flanked by UTRs, a 5’ cap and a poly-A tail. Finally, the mature mRNA can be translated into a protein by a ribosomal complex.^14,15^

Nucleotides are the basic building blocks of DNA (double-stranded molecule) and RNA (single-stranded molecule), consisting of a nitrogenous base and a five-carbon sugar (nucleoside) and a phosphate group. DNA is composed of adenine (A) and guanine (G) – purine group – and cytosine (C) and thymine (T) – pyrimidine group. On the other hand, RNA has a slight difference: thymine is replaced with uracil (U). The information within genetic material is translated by a set of rules, known as the genetic code, in which a triplet of nucleotides, also known as the codon, is read and translated into a corresponding amino acid.^14,15^ The genetic information can be modified by changing the nucleotides, causing a sequence variant, which can be classified as a transversion (substitution of a purine – two ring - for a pyrimidine - one ring - or vice versa) or a transition (substitution of a purine base to a purine or a pyrimidine base to a pyrimidine).^15^ These genetic variations may involve several nucleotides or a single one. The latter case, known as Single Nucleotide Polymorphism (SNP), is the most common type of genetic variation among humans and other species and is the main cause of phenotypic variations.^14^ An alteration in the protein coding sequence of a gene can be a non-synonymous or a synonymous substitution. A synonymous substitution results in an amino acid change and can be classified as: missense mutation (originating a codon for a different amino acid) or nonsense mutation (originating a stop codon).^14,15^ While nonsense mutations lead to a truncated protein, missense mutations make a complete protein, however the amino acid alteration may have a direct effect on protein stability and activity, conformational dynamics, cellular localization, hydrogen bonding network and pH dependance.^17^ On the other hand, synonymous substitution, known as silent mutation, does not result in an amino acid change, but it can provoke indirectly a critical impact on protein sequence and expression. Briefly, these synonymous substitutions can modify the way the transcriptional and RNA processing machinery operates, leading to alternative transcripts or different gene expression rates and protein expression levels. It should be noted that mutations not only occur in the protein coding sequence but may also occur in the intronic regions and regulatory sequences, having the same outcome as already described in synonymous substitutions.^18^

To understand gene expression, it is mandatory to also look beyond the DNA sequence. Sometimes phenotypic variations are evident, but the genotype remains unchanged, meaning that modifications in gene expression occur without involving an alteration in the DNA sequence. Epigenetics is generally accepted and defined as the study of changes in gene function that are mitotically and/or meiotically heritable and that do not entail a change in DNA sequence.^19^ This phenomenon is based on four main mechanisms which promote conformational changes in chromatin and may lead to the gene expression or repression: DNA methylation, histone modifications, chromatin remodelling and non-coding RNAs.^20^ Thus, epigenetics associated with genetics studies are of great importance to understand the molecular mechanisms and their impact in disease processes.^21^

Genomic medicine uses genetic data in order to identify individuals with higher or lower predisposition for a given condition and aims to support a more individualized clinical approach and a precision medicine strategy.^22^ In canine PD, this knowledge can potentially provide the veterinarian with the tools that can provide individualized prophylactic and therapeutic measures.^23,24^ Although these methodologies have been translated with success in human medicine (eg PST® - Periodontal Susceptibility Test for IL-1α and IL-1β gene variations), veterinary genomics is still in its infancy regarding the foundation as well as its clinical application.

The search for candidate genes allows researchers to understand the genetic basis of PD through the detection of variations that may result in changes of tissue structure or/and in response to antibodies and inflammatory mediators, conferring an increased or decreased tendency to manifest the disease.^25,26^ In contrast to Mendelian diseases, PD is not the result of a single mutation or mutation in a single gene. In fact, hundreds or thousands of genes can be associated with disease progression.^4^ Thus, variations in several genes with an important role in host-response patterns, inflammatory and immune reactions, in the scope of canine PD, such as IL1A and IL1B, IL6, IL10, LTF and TLR9, have been previously investigated.^27,31^

**Figure 1.**
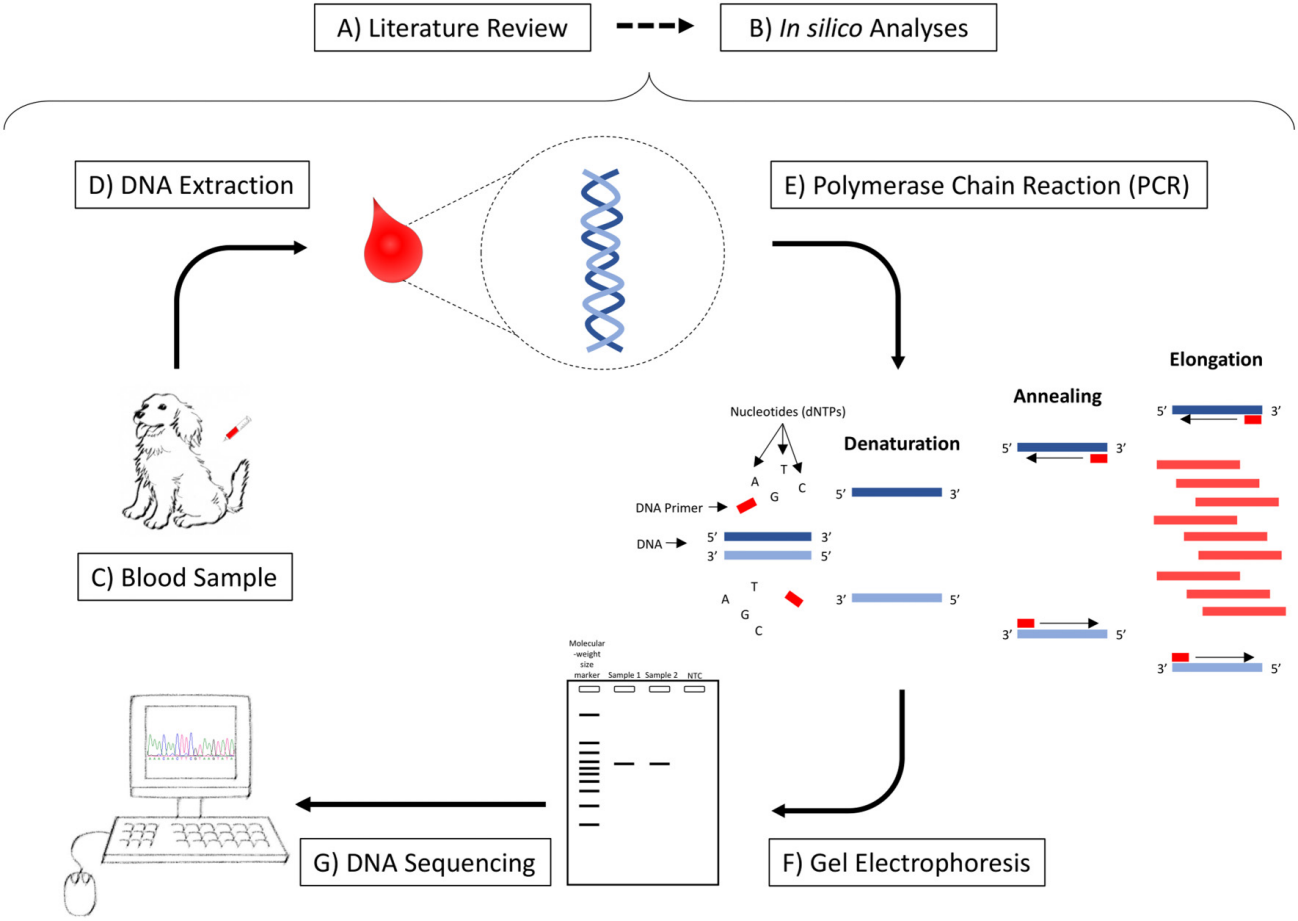
Steps required for Genomic Medicine studies. A) Literature review to select the best candidate gene; B) In silico analyses: genomic alignments among species and primers design; C) Collection of blood samples from the study population; D) DNA extraction from the blood samples; E) Amplification of a fragment of interest by PCR; F) Confirmation of the amplification of the expected fragment and contamination and primer-dimer formation through the no template control in a gel electrophoresis; G) Sequencing and identification of genetic variations.

The purpose of this manuscript is to demonstrate how genomic analysis can be applied to the study of canine PD. In this context, we describe a flowchart starting from the initial *in silico* analyses to select the candidate gene, followed by the laboratory steps and consequent characterization of the genetic variants and, finally, the identification of potential genetic variations that might be associated with clinical manifestations of PD in canine patients (Figure 1).

## Methods

In a typical case-control study, it is mandatory to present key elements of the study design in order to justify interest, its potential and the implications. Thus, the present study followed the STROBE statement for case-control studies.

The reason for the selection of the candidate gene and region of interest in the present work was its implication in one of the physiological mechanisms associated with PD. Differentiation of osteoclasts through the RANK/RANKL/OPG system pathway has already been described in the regulation of bone destruction in PD.^32^ Thus, the tumor necrosis factor receptor superfamily, member 11a, NFKB activator (TNFRSF11A) gene, also known as receptor activator of nuclear factor-kappa B (*RANK*) gene which controls the activation of osteoclastogenesis through the RANK/RANKL(receptor activator of nuclear factor NF-κB ligand)/OPG (osteoprotegerin) system, was the candidate gene selected in the present study (Figure 1A).

After choosing the candidate gene, it is essential to perform an *in silico* analysis in order to select the best region of *RANK* to be analysed, based on the similarity of the human and dog genes (Figure 1B). An alignment between the canine (Ensembl ID: ENSCAFG00000000075.4) and human (Ensembl ID: ENSG00000141655) *RANK* gene was performed using Cluster Omega software^a^, allowing the selection of a high similar sequence (exon 7 in dog) that includes an important polymorphism related to PD in humans.^33^

**Figure 2.**
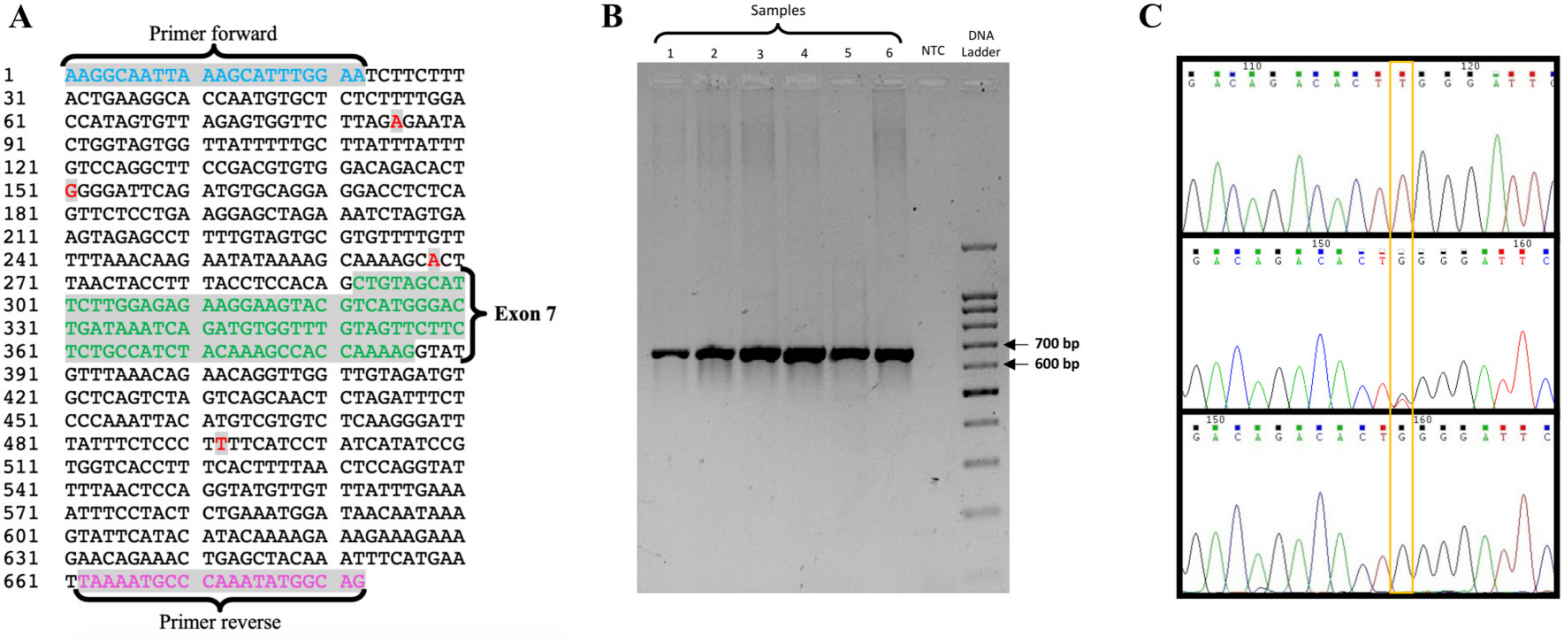
A) Nucleotide sequence of the selected fragment corresponding to exon 7 (green) and schematic representation of the genetic variations detected in this study (red), g.85A> G, g.151G> T, g.268A> G and g.492T> C, standing all of them in the intronic region. The nomenclature used in the appointment of these variations was assigned based on the polymorphism position present in the PCR fragment. B) Agarose gel showing the amplification of a fragment of 682 bp in the wells 1 to 6, the no template control (NTC) in the seventh well without the appearance of any band and the molecular-weight size marker in the eighth well. C) Electropherograms of three distinct samples evidencing the polymorphic site g.151G>T: homozygote TT on the upper electropherogram, heterozygote GT on the middle electropherogram and homozygote GG on the lower electropherogram.

Primer3Plus software^b^ was used to choose the primers for the amplification of a specific fragment from canine *RANK* exon 7. The specific primers were selected in order to amplify a 682 base pair (bp) fragment: forward – 5′-AAGGCAATTAAAGCATTTGGAA-3′ – and reverse – 5′-CTGCCATATTTGGGCATTTTA-3′ (Figure 2A).

## Study Implementation

### Clinical Examination and Biological Material Collection

90 dogs were selected from the pool of animals observed at the Veterinary Dentistry Service of the Veterinary Hospital of University of Trás-os-Montes and Alto Douro (UTAD) in association with the canine shelter Cantinho dos Animais Abandonados de Viseu. In the selection procedure, after an informed consent by the owners and following all applicable international, national, and institutional guidelines for the care and use of animals, an exhaustive medical history and a general clinical examination were performed to measure the individual health condition and to exclude other pathologies. The diagnostic criteria for PD followed the American Veterinary Dental College (AVDC) guidelines.^34^ Each tooth was evaluated individually and the total mouth periodontal score was applied to stage the PD in the studied dogs.^35^

The selected population for this study was classified according to the sex, age, weight and presence/absence of disease, following the eligibility criteria: similar feeding habits (mix of home-prepared food and commercial diets), identical mesocephalic skull type and unrelated/unfamiliar individuals, in order to decrease any source of bias. None of these animals had experienced dental preventive measures (eg tooth brushing, dental diets) or dental treatment before. The dogs analysed were mixed-breed with body weight ranging from 9-18 kg and ages varying between 2-8 years and were divided into two groups: the case group [n = 40; age range: 2-8 years (mean, 4.3); weight range: 8.4-15 kg (mean, 11.1)] and the control group [n = 50; age range: 2-5 years (mean, 3.9); weight range: 6.3-19 kg (mean, 12.2)].

Three mls of blood was collected from the external jugular vein from each dog and placed into a tube containing ethylenediaminetetraacetic acid (EDTA) (Figure 1C).

## Laboratory Work

### DNA Extraction

From the collected blood, DNA extraction was performed using the commercial QuickGene Whole Blood DNA Kit S (DB-S)^c^ and the equipment Fujifilm QG-Mini 80^d^ (Figure 1D).

### DNA Amplification by Olymerase Chain Reaction (PCR)

PCR is a technique based on the amplification of a specific DNA fragment, using two primers, which weaken the DNA segment being amplified and an enzyme, DNA polymerase, that will add the nucleotides (dNTPs), idealized in a study published in 1986 (Figure 1E).^36^ The reaction mixture is heated to denature the DNA (separate the two strands of the DNA) and then the temperature is lowered to anneal the primers into the target DNA. After annealing, the DNA polymerase enzyme adds nucleotides from the 3’ end of the primers – extension – having the target DNA strand as the template. This mixture is heated again to denature and repeat the process.

The reaction mixture contained: water (6 μL); MyTaq^TM^ HS Mix 2x^e^ (10 μL); primer forward (100 ng/μL) and reverse (100 ng/μL) (1 μL from each) and DNA from each sample (50-100 ng/μL) (2 μL). After preparation of the reaction mixture, samples were placed in the thermocycler^f^ with an optimized thermal cycle: an initial denaturation at 95 °C for 5 min followed by 40 cycles of 95 °C for 30 s, 58 °C for 30 s, 72 °C for 45 s and a final extension at 72 °C for 10 min.

### PCR Sequencing

Prior to sequencing, the PCR products were separated by agarose gel electrophoresis. This method is based on the principle that DNA has a negative charge, conferred by the phosphate group, and moves towards the positive pole. The DNA fragments migrate to different distances depending on their size (Figure 1F). Thus, this technique allows verification of PCR efficiency and confirms the amplification. The electrophoresis was performed with a 1.5% agarose gel (0.6 g of agarose; 40 mL of TBE buffer; 1.5 µL of Green Safe) at 90 V. The wells of the agarose gel were loaded with 5 µL of PCR mixture and 2 µL of DNA loading dye (10 mM Tris-HCl (pH 7.6); 10 mM EDTA; 0.005% bromophenol blue; 0.005% xylene cyanol FF; 10% glycerol) and, one of them, with the molecular-weight size marker.^g^ After electrophoresis, the gel was analysed in the transilluminator.^h^

After amplification of the DNA by PCR, primers and dNTPs that were not used in the reaction remain in the PCR mixture. In order to not interfere with the sequencing process, it is necessary to eliminate them. Thus, the PCR products were purified using Illustra ExoProStar 1-Step.^i^

DNA sequencing is a technique that determines the sequence of nucleotides in a DNA fragment. The computerized sequencing procedure uses fluorescent labeling and each base is represented by a different color (Figure 1G). All samples were bi-directionally sequenced at StabVida facilities (Lisbon, Portugal).

### Identification of Sequence Variants

Sequences were aligned, edited and analysed using the web application Clustal Omega and GeneDoc (version 2.7.000) in order to detect and validate possible genetic variations.

## Association Studies

The calculation of allele and genotype frequencies and statistical analysis was done through Statistical Package for Social Sciences (SPSS)^j^ software (version 22.0). The values obtained for the odds ratio (OR) calculation was estimated along with the 95% confidence interval (95% CI) to evaluate the association between the polymorphic genotypes/alleles of the *RANK* gene and the risk of developing PD. Moreover, adjusted ORs (controlling for age, weight and gender variables) were calculated with a logistic regression model in order to minimize potential sources of bias. The p-values were also calculated, in which p < 0.05 was considered statistically significant.

Furthermore, in order to verify if the detected genetic variations can lead to alternative splicing, an *in silico* analysis was performed through the Human Splicing Finder web application.

## Results

The aim of this research was to detect genetic variations on the canine *RANK* gene and verify a potential association to PD in a case-control study. According to the *in silico* analysis, a 682 bp fragment would be expected as confirmed by the gel (Figure 2B).

Sequencing allowed the identification of four new genetic variations in the intronic region of the fragment – g.85A>G, g.151G>T, g.268A>G and g.492T>C – as shown in Figure 2A and the variant g.151G>T was used as example in the electropherogram shown in Figure 2C. The nomenclature used in the appointment of these variations was assigned based on the polymorphism position present in the PCR fragment. The sequence data were submitted to GenBank under the accession number KR736353.

To find out whether the population is in equilibrium it was necessary to calculate the frequencies of allele and genotype observed in order to compare it with the genotype frequencies expected under the Hardy-Weinberg principle. This theorem states that allele and genotype frequencies in a population will not change from generation to generation in the absence of other evolutionary pressure.^37^ Based on the values of χ2, it was observed that for a significance level of 5% (sl = 0.05) and a number of degrees of freedom equal to 1 (df = 1), the population does not respect Hardy-Weinberg equilibrium when the χ2 value is higher than 3.841. The control group shows a χ2 value of 0.863 and the case group presents a value of 0.654. Thus, we may conclude that the population is in equilibrium, according to Hardy-Weinberg equilibrium principles.

The statistical analysis was performed in order to observe the differences in allele and genotype distributions between the control group and the case group. Table 1 contains the values obtained for the OR calculation which was estimated along with the 95% confidence interval (95% CI). These values evaluate the association between the polymorphic genotypes of the *RANK* gene and the risk of developing PD. The p-value was also calculated and all the correlations in which p < 0.05 were statistically significant. The statistical results obtained showed that there were no significant differences between the control group and the case group with PD. After the combination of the dominant homozygous genotype with the heterozygous, statistically significant differences were not observed, not even when adjusted for sex, age and weight. The same was true after the recessive homozygous genotype was combined with the heterozygous genotype, including after the adjustment. There are still no significant differences for individuals with polymorphic alleles, even when adjusted for sex, age and weight. Despite the four variations of the *RANK* gene discovered in dogs, none of the variants were found to have a statistically relevant effect on either the likelihood of developing PD or on the severity of PD in those dogs with clinical disease.

**Table 1.** Genotype Frequency and Odds Ratio of all Polymorphisms in the Case and Control Groups.

Polymorphism	Genotype/Alleles	Case Group (n = 40) n (%)	Control Group (n = 50) n (%)	OR (95% CI)	P value*	Adjusted OR§ (95% CI)	P value*
**g.85A>G**	AA	13 (32.5)	15 (30.0)				
	AG	22 (55.0)	23 (46.0)				
	GG	5 (12.5)	12 (24.0)				
	GG+AG	27 (43.5)	35 (56.5)	0.89 (0.363-2.182)	0.799	0.69 (0.264-1.808)	0.450
	AA+AG	35 (87.5)	38 (76.0)	2.211 (0.707-6.911)	0.173	3.107 (0.853-11.315)	0.086
	A	48 (60.0)	53 (53.0)	Ref.		Ref.	
	G	32 (40.0)	47 (47.0)	0.752 (0.415-1.363)	0.347	0.620 (0.324-1.186)	0.148
**g.151G>T**	GG	13 (32.5)	15 (30.0)				
	GT	22 (55.0)	23 (46.0)				
	TT	5 (12.5)	12 (24.0)				
	TT+GT	27 (43.5)	35 (56.5)	0.89 (0.363-2.182)	0.799	0.69 (0.264-1.808)	0.450
	GG+GT	35 (87.5)	38 (76.0)	2.211 (0.707-6.911)	0.173	3.107 (0.853-11.315)	0.086
	G	48 (60.0)	53 (53.0)	Ref.		Ref.	
	T	32 (40.0)	47 (47.0)	0.752 (0.415-1.363)	0.347	0.620 (0.324-1.186)	0.148
**g.268A>G**	AA	13 (32.5)	15 (30.0)				
	AG	22 (55.0)	23 (46.0)				
	GG	5 (12.5)	12 (24.0)				
	GG+AG	27 (43.5)	35 (56.5)	0.89 (0.363-2.182)	0.799	0.69 (0.264-1.808)	0.450
	AA+AG	35 (87.5)	38 (76.0)	2.211 (0.707-6.911)	0.173	3.107 (0.853-11.315)	0.086
	A	48 (60.0)	53 (53.0)	Ref.		Ref.	
	G	32 (40.0)	47 (47.0)	0.752 (0.415-1.363)	0.347	0.620 (0.324-1.186)	0.148
**g.492T>C**	TT	13 (32.5)	15 (30.0)				
	TC	22 (55.0)	23 (46.0)				
	CC	5 (12.5)	12 (24.0)				
	CC+TC	27 (43.5)	35 (56.5)	0.89 (0.363-2.182)	0.799	0.69 (0.264-1.808)	0.450
	TT+TC	35 (87.5)	38 (76.0)	2.211 (0.707-6.911)	0.173	3.107 (0.853-11.315)	0.086
	T	48 (60.0)	53 (53.0)	Ref.		Ref.	
	C	32 (40.0)	47 (47.0)	0.752 (0.415-1.363)	0.347	0.620 (0.324-1.186)	0.148

*Chi-square test or Fisher's two-tailed exact test, when applicable; ^§^OR adjusted for age, weight and sex.

## Discussion

The importance of canine PD in veterinary medicine is unquestioned due to its widespread prevalence^1,2,11^ and the numerous local and potentially systemic consequences on health. Although not extensively characterized in dogs, the research in human patients has described a relationship between PD and renal, hepatic, pulmonary and cardiac diseases, osteoporosis, adverse pregnancy effects and diabetes mellitus.^4,6^

The knowledge of this condition in dogs could be also useful in the study of human PD, due to the recognized interest in canine species as a model for human PD and periodontal regeneration research.^5,38^

The PD aetiology is multifactorial and can be analysed from different perspectives.^39^ Firstly, by detecting the presence of microbial populations with potential for inflammation; secondly, by examining the level of systemic health condition and environmental factors that modify the host response in protective or destructive pathways; and finally PD can be analysed at the host level, based on the genetic factors that can predispose to or protect from PD.

The progression of PD instigates a route of alveolar bone resorption by osteoclasts, and degradation of ligament fibers by matrix metalloproteinases and the formation of granulation tissue.^4^

The mechanism of osteoclastogenesis is known to be controlled both directly, through insulin-like growth factor 1 (IGF-1) receptor present on osteoclasts, and by upregulating RANKL, a membrane-bound protein expressed primarily on the surface of osteoblasts and bone marrow stromal cells and secreted in a soluble form by activated T lymphocytes.^40^ RANKL binds to RANK on the surface of pre-osteoclasts, enabling the recruitment of the adapter protein TRAF6, activating nuclear factor kB, resulting in increased transcription of genes involved in osteoclastogenesis.^41,42^ On the other hand, osteoprotegerin (OPG) is a soluble receptor normally produced and secreted by bone marrow stromal cells and osteoblasts whose biological effects on bone cells include the inhibition of osteoclast differentiation, suppression of osteoclasts matrix activation and apoptosis.^43^ Thus, OPG protects against excessive bone reabsorption due to its link with RANKL and the osteoclastogenesis is suppressed by the reduction of interaction between RANKL and RANK.^44^ As uncontrolled osteoclastic activity can produce deleterious effects, the biologic activity of RANKL is tightly counter regulated by OPG.^42^

Osteoclast differentiation through the RANK/RANKL/OPG system seems to be a pathway with implications in the regulation of bone destruction in PD patients.^32^ SNPs in the *RANK* gene have been implicated in different human diseases, including Paget's Disease, rheumatoid arthritis and osteoporosis.^33,45,47^

The present work focused on the characterization of *RANK* gene polymorphisms and the canine PD. The results showed that a region of this gene seems to have potential interest due to the presence of rs1805034 SNP, identified as being caused by an allelic variation of C/T responsible for the substitution of a valine for an alanine (V192A).^33^

The present study was conducted in order to analyse the region of particular importance of the *RANK* gene. We were able to identify four new genetic variations designated as: g.85A>G, g.151G>T, g.268A>G and g.492T>C, located in the intronic region of the fragment, as shown in Figure 2A. None of these genetic variations coincided with those described in humans. Although the positions may not necessarily be the same when comparing two different species, regions with genetic variations have a higher tendency to remain as polymorphic regions among species.^48^

The population under study is in equilibrium with the principles of Hardy-Weinberg, meaning that the observed values are close to the expected values and we may consider it a homogeneous population. Statistical analysis was performed in order to observe the differences in allele and genotype distributions between the control group and the case group. In Table 1, the statistical results showed there are no significant differences between the control group and the case group with PD. After the combination of the dominant homozygous genotype with the heterozygous, statistically significant differences were not observed (OR = 2.211; 95% CI: 0.707-6.911; p = 0.173), not even when adjusted for sex, age and weight (OR = 3.107; 95% CI: 0.853-1.1315; p = 0.086). The same was true after recessive homozygous genotype combination with the heterozygous genotype (OR = 0.89; 95% CI: 0.363-2.182; p = 0.799), including after the adjustment (OR = 0.69; 95% CI: 0.264-1.808; p = 0.450). There are still no significant differences for individuals with polymorphic alleles (OR = 0.752; 95% CI: 0.415-1.363; p = 0.347), even when adjusted for sex, age and weight (OR = 0.620; 95% CI: 0.324-1.186; p = 0.148).

All genetic variations identified in this study were located at an intronic region and thus could not change the amino acid in the final protein. For many years, it was considered that the introns were simple nucleotide sequences that should be removed in order to enable the correct gene expression, but recent studies have shown that SNPs present in the intronic regions are also important because they can change the setting of the gene transcription. Several genes can generate multiple messenger RNA (mRNA) variants and protein isoforms through a process designated as alternative splicing.^49^ According to Human Splicing Finder software, the g.85A>G, g.151G>T and g.492T>C variations may create or modify splicing silencer and/or splicing enhancer sites and can lead to a potential alteration of splicing. However, in the case of g.268A>G mutation, no significant splicing motif alteration was detected, which means that this genetic variation has probably no impact on splicing. Although it is not possible to associate any variation identified in this work with canine PD, the genetic variations, which are present in this gene, may be important if they affect the level of expression of the *RANK* gene.

The development of new diagnostic strategies is supported by an initial identification and characterization of polymorphisms. The genetic basis of human PD has gone from simple experimental evidence to direct effects on early diagnosis and new therapeutic strategies.^50^ Multiple candidate genes are initially envisaged and some of them demonstrate potential application in clinical practice. *IL1* and *IL6* genes have already been used for the development of two diagnosis kits in humans.^51,52^ In veterinary medicine there is still a long way to go, although recent studies showed very interesting results for *IL1A* and *TLR9* genes, with IL1A/1_g.388C allele possibly associated with a decreased PD risk and IL1A/1_g.521A allele conferring an increased risk and the rs22882109 and rs22882111 polymorphisms in *TLR9* associated with the predisposition to PD.^27,28^ However, considering that the polymorphisms may have different effects in different ethnic groups, in order to validate the results and clarify the role of the novel genetic variations identified in the case/control approaches, future studies should use specific dog breeds.

An extensive genetic characterization of canine PD allows early identification of animals susceptible to develop the disease, helping to design more effective and personalized prophylactic and therapeutic measures.

## Conclusion

In the present work, through a case-control study performed in dogs, novel *RANK* gene polymorphisms were identified and analysed in order to evaluate their possible association with susceptibility to PD.

To the best of our knowledge, this is the first study conducted in dogs that intends to establish a relationship between polymorphic variations in the *RANK* gene of individuals predisposed to PD, and although this association was not made, it is important to develop new investigations in order to clarify and validate the genetic variations observed in this study. Moreover, in genomic medicine studies, reporting these results is very important, in order to prevent other researchers from reanalzsing this gene region and it increases information in this area which is still in early stages of development. It is also important that further research should involve other polymorphic variations in order to elucidate the role of this gene in PD. Future studies on the regulation of expression and analysis of the existing transcripts concerning animals with and without disease will be essential to understand these biological mechanisms. Considering the rapid development of new technologies and the increase in research studies in this domain, future development of early diagnostic strategies look very promising.
